# Human Orthobunyavirus Infections, Tefé, Amazonas, Brazil

**DOI:** 10.1371/currents.outbreaks.7d65e5eb6ef75664da68905c5582f7f7

**Published:** 2018-03-22

**Authors:** Felipe Gomes Naveca, Valdinete Alves Nascimento, Victor Costa Souza, Regina M.P. de Figueiredo

**Affiliations:** Laboratório de Ecologia de Doenças Transmissíveis na Amazônia, Instituto Leônidas e Maria Deane - Fiocruz Amazônia, Manaus, Amazonas, Brazil; Laboratório de Ecologia de Doenças Transmissíveis na Amazônia, Instituto Leônidas e Maria Deane - Fiocruz Amazônia, Manaus, Amazonas, Brazil; Laboratório de Ecologia de Doenças Transmissíveis na Amazônia, Instituto Leônidas e Maria Deane - Fiocruz Amazônia, Manaus, Amazonas, Brazil; Gerência de Virologia, Fundação de Medicina Tropical Doutor Heitor Vieira Dourado, Manaus, Amazonas, Brazil

**Keywords:** arbovirus, Brazil, dengue, infectious diseases, Mayaro, Oropouche, Orthobunyavirus

## Abstract

**Introduction:**

Several orthobunyaviruses are important arthropod-borne pathogens, responsible for a variety of diseases in humans, from acute febrile illness to encephalitis.

**Methods:**

We collected serum samples from a series of dengue suspected cases in Tefé, a mid-size city located in the interior of the Amazonas state, Brazil. Viral RNA extraction was performed, and specimens were tested for dengue virus using RT-PCR. Thirty dengue negative samples were further tested for Mayaro virus (MAYV) and Oropouche virus (OROV) using an RT-qPCR protocol previously described. Positive samples were characterized by MegaBLAST analysis over the entire nucleotide collection of the main public databases, and also by maximum likelihood phylogenetic reconstruction of the S genome segment.

**Results:**

We detected nine OROV or OROV-like positive cases among 30 patients reporting fever and headache, as the most common symptoms. The closest nucleotide sequence returned from the MegaBLAST analysis belongs to an OROV isolated in Peru 2008. Moreover, all Tefé samples grouped in the same clade with the OROV reference sequence and other closely-related OROV-like viruses.

**Discussion:**

Dengue viruses are still the most important arbovirus worldwide, causing hundreds of millions of infections every year. Nonetheless, other arboviruses like chikungunya virus, Zika virus, and yellow fever virus have emerged in the last few years and are now a public health concern in several countries. OROV is believed to have caused more than 500,000 febrile infections in Brazil over recent decades. Therefore, the results described in this study strengthen that this arbovirus, and its closely-related recombinants, should be under continuous surveillance, at least in the endemic countries of Latin America.

## REPORT

Between April and June 2015, professionals of the health surveillance system at Tefé (03° 21' 14" S; 64° 42' 39" W), a mid-size city located in the interior of the Amazonas state, Brazil, collected serum samples from a series of dengue suspected cases. The specimens were obtained between the first and second day after symptom onset and sent to Fundação de Medicina Tropical – Dr. Heitor Vieira Dourado (FMT-HVD) at Manaus, Amazonas, Brazil for dengue virus (DENV) testing using RT-PCR 1.

Thirty DENV negative samples were randomly selected and sent to Instituto Leônidas e Maria Deane (ILMD), Fiocruz Amazônia, to investigate other Amazonian endemic arboviruses 2^,^^3^^,^^4^^,^^5^. At ILMD, samples were tested using a multiplexed reverse transcription real-time PCR protocol for Oropouche, and Oropouche-like viruses, (OROV, *Peribunyaviridae* family, genus *Orthobunyavirus*), targeting the S segment; as well as for Mayaro virus (MAYV, *Togaviridae* family, *Alphavirus* genus) aiming the NSP1 coding region 6. All patients gave written informed consent, and the principal investigators anonymized the participants’ data. The ethics committee of FMT-HVD approved the study under the registration number 700.915

We detected nine OROV or OROV-like positive cases (TF031; TF036; TF070; TF081; TF086; TF087; TF100; TF101; TF103), seven males and two females, with a mean age of 36.2 years (range 8 - 63), living in either rural or urban areas of Tefé. Regarding the clinical symptoms, all nine patients reported fever and headache; two patients also reported myalgia and arthralgia and one showed retro-orbital pain. Therefore, these patients presented non-specific symptoms that could easily lead to dengue misdiagnosis, if no specific laboratory testing were applied.

To further confirm these results, all nine samples were submitted to an RT-PCR protocol targeting the S segment of OROV/OROV-like viruses using primers OROV_S_134F 5'- CGGACAAGTGCTCAATGCTG -3' and OROV_S_734F 5'- GTCAATTCCGAATTGGCGCA - 3', generating a 601bp amplicon. These products were used for capillary nucleotide sequencing in an ABI3130 genetic analyzer, installed at the ILMD genomics platform. After removal of the primers, the final 558bp sequence for each sample was used for a multiple alignment with MAFFT^7^, embedded in Geneious Software 10.2.2 8. The orthobunyavirus sequences obtained in this study were highly conserved among them, as should be expected for any arbovirus samples collected from the same area, in a small-time frame. Only one nucleotide variation was observed in the sample TF86, a silent mutation (Transversion: T / A) in position 386 related to the OROV reference sequence for segment S NC_005777.

We also conducted a BLAST analysis 9 over the consensus nucleotide sequence, using the MegaBLAST algorithm 10. Only orthobunyavirus sequences were retrieved among the first 100 hits with the higher score and lower E-values. The closest nucleotide sequence returned from the MegaBlast analysis belongs to an Oropouche virus isolated in Peru 2008 (strain TVP-19256/IQE-7894 - KP795086) sharing 98% similarity, with 545/558 identical sites.

Recently, the International Committee on Taxonomy of Viruses (ICTV) made several changes in virus taxonomy, comprising the creation of the* Bunyavirales *order*. *This new order contains eight new viral families (*Feraviridae*;* Fimoviridae*;* Hantaviridae*;* Jonviridae*;* Nairoviridae*;* Phasmaviridae*;* Phenuiviridae *and* Tospoviridae*) and one family that was renamed:* Peribunyaviridae*, formerly* Bunyaviridae. *Two genera are included in the* Peribunyaviridae *family, genus* Herbevirus *and genus* Orthobunyavirus *11.

Currently, ICTV recognizes 48 species in the *Orthobunyavirus* genus (ICTV Master Species List 2016 v1.3 - available at https://talk.ictvonline.org/files/master-species-lists/m/msl/6776/download). According to the ICTV Virus Metadata Repository (version November 8, 2017, available at https://talk.ictvonline.org/taxonomy/vmr/m/vmr-file-repository/7019), among those 48 orthobunyaviruses species, 35 have sequence data deposited in GenBank regarding the S segment. Thus, we used the PhyML software 12 embedded in Geneious Software 10.2.2, for maximum likelihood phylogenetic reconstruction with the ICTV recognized orthobunyavirus species; our data from the Tefé samples and six OROV-like viruses marked with a star (*). The phylogenetic tree was edited with FigTree v1.4.3, available at http://tree.bio.ed.ac.uk/software/figtree/.

All Tefé samples grouped in the same clade with the species *Oropouche orthobunyavirus* (RefSeq NC_005777) and the closely-related OROV-like viruses (Utinga virus, Utive virus, Madre de Dios virus, Iquitos virus and Perdões virus). One exception was observed for the Facey’s Paddock virus, another OROV-like virus isolated initially in Australia 1974, which grouped with the Manzanilla orthobunyavirus isolated in Vietnam, 2004 (Fig 1).

**Maximum likelihood tree of the genomic S segment of orthobunyaviruses.**
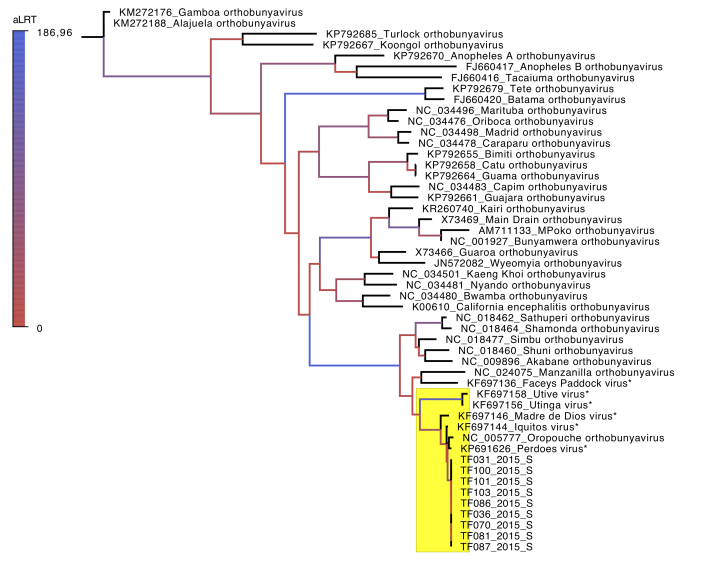
All ICTV recognized orthobunyavirus species with available genomic data for the S segment were used for phylogenetic reconstruction, together with Tefé samples and other OROV-like viruses. The clade containing the Tefé samples, grouped with the species *Oropouche orthobunyavirus*, and the closely-related OROV-like viruses, is highlighted in yellow. Branches are colored according to the aLRT support.

Annually, dengue viruses cause hundreds of millions of febrile infections, of widely varying intensity, in humans throughout the tropics and sub-tropics. Oropouche fever, caused by Oropouche virus (OROV) is less well studied but is believed to have caused more than 500,000 febrile infections in Brazil over recent decades 13. Such cases could easily be clinically misdiagnosed for dengue infections. Moreover, OROV is a typical orthobunyavirus possessing a triple-segmented RNA genome which readily undergoes genetic reassortment with related orthobunyaviruses 14, potentially resulting in genetically distinct recombinant variants of OROV 15^,^^16^^,^^17^^,^^18^. Consequently, further molecular studies are currently being undertaken to determine the extent to which infections due to OROV and/or OROV-related recombinants (for example, Perdões virus), are being mistakenly clinically diagnosed as being caused by dengue viruses.

## Funding

FGN is funded by Fundação de Amparo à Pesquisa do Estado do Amazonas - FAPEAM (www.fapeam.am.gov.br, call 001/2013 - PPSUS / 062.00656/2014 and call 001/2014 - PROEP / 062.01939/2014); Conselho Nacional de Desenvolvimento Científico e Tecnológico (http://www.cnpq.br, grant 440856/2016-7) and Coordenação de Aperfeiçoamento de Pessoal de Nível Superior (http://www.capes.gov.br, grants 88881.130825/2016-01 and 88887.130823/2016-00) call MCTIC/FNDCT -CNPq / MEC-CAPES/ MS-Decit 14/2016 - Prevenção e Combate ao vírus Zika. RMPF is funded by Fundação de Amparo à Pesquisa do Estado do Amazonas - FAPEAM (www.fapeam.am.gov.br, call 001/2013 - PPSUS). The funders had no role in study design, data collection and analysis, decision to publish, or preparation of the manuscript.

## Competing Interests

The authors have declared that no competing interests exist.

## Data Availability

All partial S segment orthobunyavirus sequences are available as a FASTA file at https://doi.org/10.6084/m9.figshare.5458342.v1/.

## Corresponding Authors

Dr. Felipe Gomes Naveca (felipe.naveca@fiocruz.br) and Dr. Regina Maria Pinto de Figueiredo (figueiredormp@yahoo.com.br).
